# Litter Species Composition and Topographic Effects on Fuels and Modeled Fire Behavior in an Oak-Hickory Forest in the Eastern USA

**DOI:** 10.1371/journal.pone.0159997

**Published:** 2016-08-18

**Authors:** Matthew B. Dickinson, Todd F. Hutchinson, Mark Dietenberger, Frederick Matt, Matthew P. Peters

**Affiliations:** 1 US Forest Service, Northern Research Station, Delaware, OH, 43015, United States of America; 2 US Forest Service, Forest Products Laboratory, Madison, WI, 53726, United States of America; University of the Chinese Academy of Sciences, CHINA

## Abstract

Mesophytic species (esp. *Acer rubrum*) are increasingly replacing oaks (*Quercus* spp.) in fire-suppressed, deciduous oak-hickory forests of the eastern US. A pivotal hypothesis is that fuel beds derived from mesophytic litter are less likely than beds derived from oak litter to carry a fire and, if they do, are more likely to burn at lower intensities. Species effects, however, are confounded by topographic gradients that affect overstory composition and fuel bed decomposition. To examine the separate and combined effects of litter species composition and topography on surface fuel beds, we conducted a common garden experiment in oak-hickory forests of the Ohio Hills. Each common garden included beds composed of mostly oak and mostly maple litter, representative of oak- and maple-dominated stands, respectively, and a mixture of the two. Beds were replenished each fall for four years. Common gardens (N = 16) were established at four topographic positions (ridges, benches on south- and northeast-facing slopes, and stream terraces) at each of four sites. Litter source and topographic position had largely independent effects on fuel beds and modeled fire dynamics after four years of development. Loading (kg m^-2^) of the upper litter layer (L), the layer that primarily supports flaming spread, was least in more mesic landscape positions and for maple beds, implying greater decomposition rates for those situations. Bulk density in the L layer (kg m^-3^) was least for oak beds which, along with higher loading, would promote fire spread and fireline intensity. Loading and bulk density of the combined fermentation and humic (FH) layers were least on stream terrace positions but were not related to species. Litter- and FH-layer moistures during a 5-day dry-down period after a rain event were affected by time and topographic effects while litter source effects were not evident. Characteristics of flaming combustion determined with a cone calorimeter pointed to greater fireline intensity for oak fuel beds and unexpected interactions between litter source and topography. A spread index, which synthesizes a suite of fuel bed, particle, and combustion characteristics to indicate spread (vs extinction) potential, was primarily affected by litter source and, secondarily, by the low spread potentials on mesic landscape positions early in the 5-day dry-down period. A similar result was obtained for modeled fireline intensity. Our results suggest that the continuing transition from oaks to mesophytic species in the Ohio Hills will reduce fire spread potentials and fire intensities.

## Introduction

**Fig 1:**
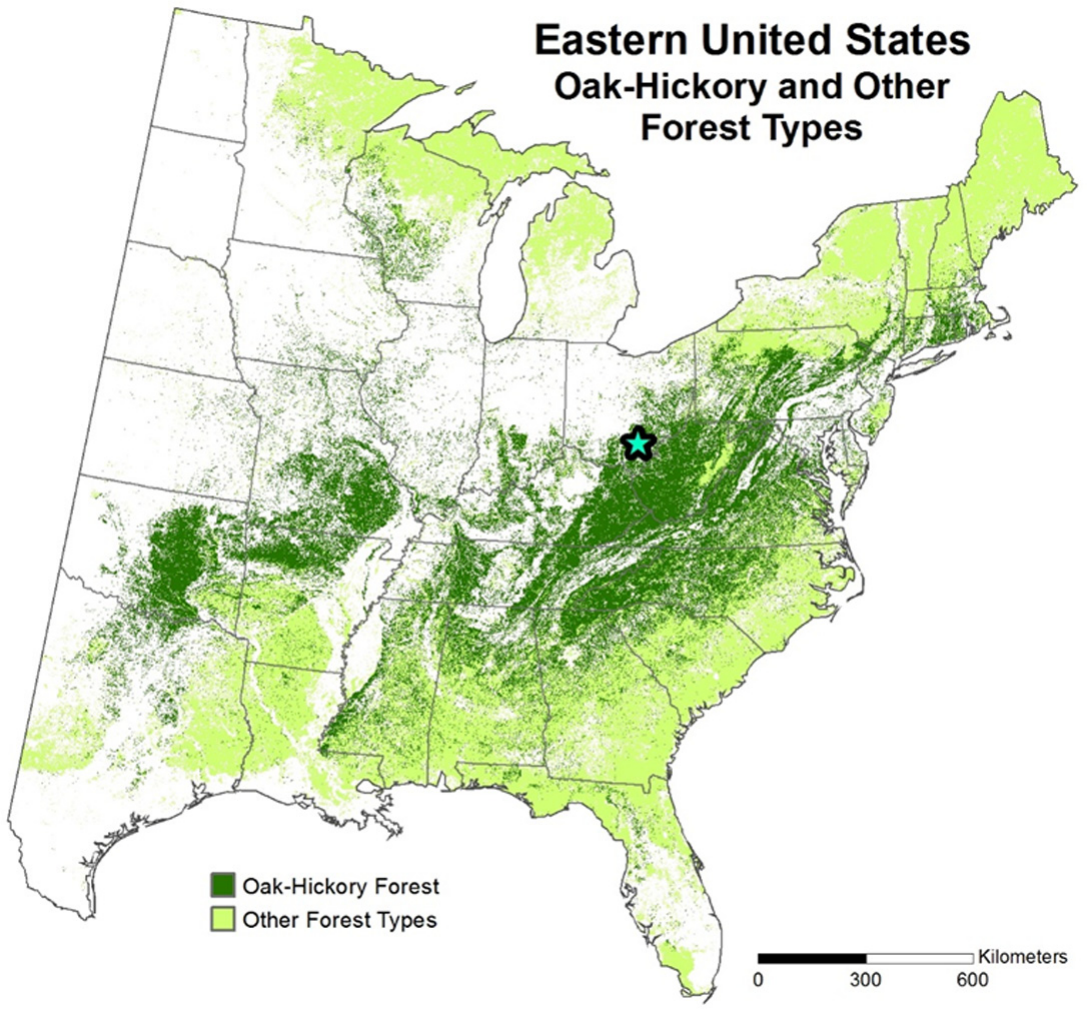
The distribution of oak-hickory forests (also known as the central hardwoods) in the eastern USA. Ruefenacht *et al’s* [5] derived forest types from US Forest Service’s Forest Inventory and Analysis program tree species composition data. The “other” category is a combination of their forest types that may include oak species, but would often differ substantially in species composition and structure from the Vinton Furnace Experimental Forest (VFEF, indicated by a star) where this experiment was conducted. Ruefenacht *et al’s* maps and underlying data are in the public domain.

The “ecology of fuels” concept emphasizes the critical role that fuels play in mediating feedbacks between fire and vegetation [1]. Fuels, along with weather and terrain, regulate fire behavior that influences vegetation composition through differential tree species resistance and resilience to fire-caused injury [2]. In turn, changes in vegetation composition influence fuel characteristics [3–4]. Understanding the dynamics of fuel beds dominated by leaf litter is key in oak-hickory forests in the eastern USA (Fig 1, based on [5]), a widespread forest type (also known as the central hardwoods) in which oaks (*Quercus* spp.) are often abundant because of climate and relatively frequent historical fires [6–7]. Overstory tree species composition is a key factor in fuel variability because trees produce litter of contrasting characteristics that affects a fuel bed’s decomposition dynamics [8], ability to carry a fire [9–10], and combustion characteristics [4, 11].

In oak-hickory forests, striking are the “fluffy” (low bulk density, kg m^-3^) and deep (high loading, kg m^-2^) fuel beds generated by a white (*Quercus alba*, *Q*. *prinus*), black (e.g., *Q*. *velutina*,), and red oak (*Q*. *rubra*, *Q*. *coccinea*) overstory [12] relative to the more compact beds that develop from overstory trees of other genera such as the “mesophytic” maples (*Acer rubrum*, *A*. *saccharum*), tulip poplar (*Liriodendron tulipifera*), and American beech (*Fagus grandifolia)*[6]. Central hardwoods oak species’ particles pack less densely because individual leaves are large [13] and tend to curl upon drying [14]. Beds with high loading (kg m^-2^) and low bulk density (kg m^-3^) promote fire spread and high heat-release rates. During the current fire suppression era [6], red maple (*Acer rubrum*) in particular has increased dramatically in abundance in oak-hickory forests [15–16] while many oak species, particularly on more mesic sites, have exhibited poor regeneration in increasingly closed-canopy forests [17]. Prescribed fire can be a highly effective tool to improve oak regeneration and sustain oak forests [18]. The same oaks that produce fire-promoting fuel beds are resistant and resilient to fire because thick bark protects mature trees from fire injury and the root-dominant growth of oak seedlings enables vigorous root collar sprouting after stems are killed by fire [19]. It has been hypothesized that the expansion of maple and the resulting “forest mesophication” process [7] will make the use of prescribed fire more difficult because litter beds dominated by maple foliage are less likely to carry a fire, particularly an intense one, than oak fuel beds. The correlation between fire-promoting litter characteristics and resistance to fire injury in oak-hickory forests is similar to southeastern ecosystems where fire-promoting litter characteristics are also correlated with resistance to fire in oaks and pines [11].

Differences among oaks and mesophytic tree species in leaf dimensions and shape combine with differences in leaf chemistry, litter drying, and rates of litter decomposition to determine fuel bed characteristics and combustion. Leaf chemistry mediates litter decomposition dynamics where high lignin to cellulose ratios [20] and high lignin to nitrogen ratios inhibit decomposition [21–22] while high contents of calcium [22] and soluble carbon compounds [23] promote decomposition. Red maple litter was shown to have higher decomposition rates than chestnut oak in Blair *et al*. [24]. Aber and Melillo [25] found that chestnut oak, scarlet oak, and white oak had higher initial lignin and lignin:nitrogen ratios than red maple and tulip poplar and would, thus, be expected to exhibit slower decomposition rates than those mesophytic species. Mixtures of litter from different species, as compared to litter from a single species, may have non-additive effects on microbial communities [24–26] and result in higher decomposition rates [8]. Decomposed litter becomes enriched in lignin, which is a strong determinant of heat release, lignin being more thermally stable than cellulose [27], leading to more char formation [28], and, thereby, promoting glowing and smoldering (non-flaming) combustion. Kreye *et al*. [29] showed that litter from mesophytic species generally exhibited slower drying rates than litter of pyrophytic species, including large-leaved oaks characteristic of oak-hickory forests.

Many parts of the central hardwoods region are characterized by dissected terrain which complicates our understanding of species effects on fuel beds and fire behavior because topographic gradients affect not only species distributions and abundances [30] but also the environment in which litter beds develop and fires occur. In the Ohio hills, oaks were found to be most abundant and to obtain higher basal areas on drier landscape positions more exposed to solar radiation (e.g., ridges and slopes with southerly aspect), tulip poplar and black cherry importance was greatest on less-exposed mesic sites, and red maples were found to be more general in their tolerances [31]. In turn, fires in the Ohio Hills were more intense on south-facing than north-facing slopes, causing greater tree mortality and canopy openness there and favoring oak dominance in the understory [32]. Boerner [33] described a feedback cycle for central hardwoods litter beds where nutrient rich litter falling from mixed mesophytic stands growing on topographically wetter and more nutrient-rich soils on north-facing slopes resulted in high rates of decomposition in contrast to low-nutrient litter falling from oak-dominated stands on drier and less fertile ridges and south-facing slopes. Generally, decomposition rates have been found to be greatest when litter beds are warmer and more moist [22, 34–36], conditions mediated by topographic gradients in insolation and soil moisture. Stottlemyer *et al*. [37] and Waldrop *et al*. [38] found topographically-related variation in fuels in dissected terrain in the southern Appalachians though differences were likely reduced by covariation of productivity and decomposition rates.

Despite the appeal of the fuel mesophication hypothesis and the examination it has received [7], the effects of species composition on fuels and fire behavior are potentially confounded in dissected terrain by the effects of topographic variability in the conditions that govern fuel bed development, moisture dynamics, and fire dynamics. In this paper, we use data from a common garden experiment in an attempt to separate the effects of litter source (oak-dominated, maple-dominated, and a mixture) from those of topography on fuel bed structure, thermochemistry, moisture dynamics, laboratory combustion, and modeled fire spread and fireline intensity. Beds composed of oak- and maple-dominated litter or a mixture of the two (the common garden) were replenished each fall for four years. Common gardens were established in mature forest at four topographic settings at each of four sites. The topographic settings covered a wide range of solar radiation and hydrologically-determined soil moisture conditions. Our overarching question is: What is the relative importance of litter source and topography on fuel properties and fire behavior? As part of our answer to this question, we explore how fuel bed characteristics, particle properties, and moisture dynamics integrate to affect the modeled propensity of fuel beds to carry a fire [9–10] and affect fireline intensity (kW m^-1^).

## Methods

### Study site and common garden experimental design

**Fig 2:**
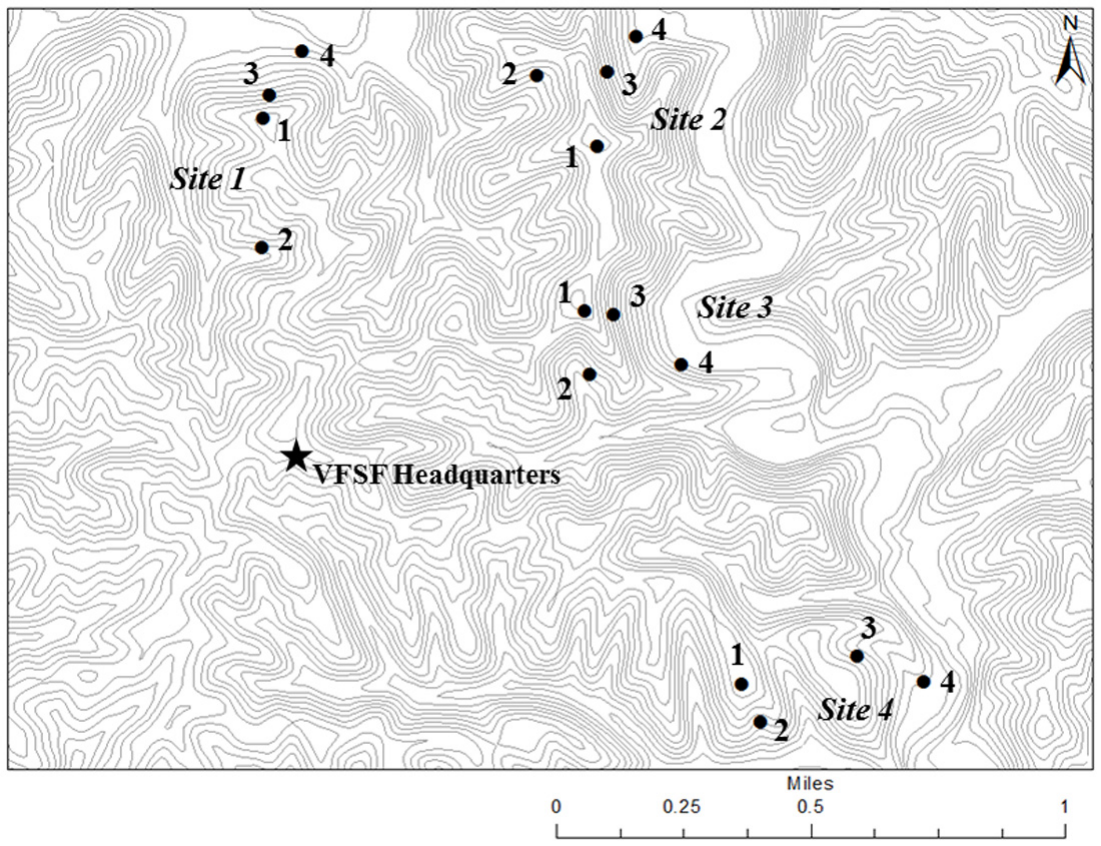
Common garden locations. Common gardens were allocated to ridge tops (1), south benches (2), northeast benches (3), and stream terraces (4) at four sites within the Vinton Furnace Experimental Forest in the Ohio Hills. Lines of equal elevation are at 20 meter intervals.

The study was conducted on the hilly and unglaciated Allegheny Plateau at the Vinton Furnace Experimental Forest (VFEF, Lat. 39°11’ N, long. 32°22’ W) in southeastern Ohio (Figs 1 and 2). The VFEF is contained within the Raccoon Ecological Management Area and the REMA Research Advisory Committee provided permission to conduct the study. At each of four sites, four landscape positions were chosen for common gardens; these were ridgetops, benches on south-facing slopes (south benches), benches on north to eastern-facing slopes (northeast or NE benches hereafter), and stream terraces at the base of north to east-facing slopes. On hillslopes, it was necessary to establish the common gardens on relatively level benches to avoid the down-slope movement of litter [39]. The order of positions listed above was expected to follow a co-gradient in increasing soil moisture and declining solar radiation.

Weather stations (Onset Computer Corporation) on 2 m tripods were established at each common garden location and instruments were checked monthly from August 2007 through May 2010. Hourly average wind and peak gust speed (averaged over each hour), solar radiation, air temperature, and soil moisture were monitored. Instruments were recalibrated or replaced yearly as needed. To best reflect conditions under which biotic processes were active, only data from when air temperatures were above 5°C were used in analyses. All variables were averaged over the measurement period for analysis. As a complement to measured data, we modeled annual solar radiation and a topographic moisture index (TMI) for the common garden locations using a Geographic Information System and a digital elevation model [31]. Solar radiation was calculated daily at 3 hour intervals and diffuse radiation and transmissivity parameters were given unique values for each month based on results from Dyer [40]. Solar radiation used for analysis was the yearly sum of solar energy (MJ m^-2^). TMI was calculated using a modified version of the Quinn *et al*. [41] equation, ln(*a*/tan(β)), based on suggestions from Sørensen *et al*. [42] where *a* is an infinite directional flow accumulation [43] in m^2^ and β is slope of the landscape in radians. We explored co-variation of environmental variables modeled and measured at each common garden location using factor analysis (PROC FACTOR) in SAS [44].

At each common garden in the fall of 2006, six 2x3 m plots were cleared of forest floor material down to mineral soil and woody stems were clipped at ground level. Then, beginning in November 2006 and ending in November 2009, 0.5 kg m^-2^ (dry mass) of three kinds of freshly-fallen litter was spread on each plot. The loading we used was the average for litter from a range of published studies (e.g., [45–47]) and our unpublished data. The beds were predominantly maple or oak litter or a mixture of the two kinds of litter (each contributing 0.25 kg m^-2^) (Table 1). Litter was collected shortly after leaf fall in all years from three oak dominated stands and three stands dominated by maples. To ensure low representation by oaks in the maple litter collections, we took advantage of the phenological difference of early maple leaf fall relative to oak. Litter was stored under a roof and allowed to dry to ambient conditions. The correct mass of litter at ambient conditions for each type of bed was calculated from litter moisture content determined from sub-samples. Litter for each bed was weighed and bagged individually for distribution to common gardens. In November 2008, in order to determine the composition of the leaf sources, we took a single “grab” sample (a large handful) from each bag of litter that was to be placed on each plot (n = 96). The samples were air dried for approximately two weeks, separated by species group (oak, maple, other species, and unidentifiable), and weighed. The unidentifiable class was largely composed of leaf fragments.

**Table 1:** The species composition by mass of litter collected in fall 2008 and re-distributed to common garden fuel beds.

Litter type	% oak^1^	% maple^2^	% other species^3^	% unidentified
Oak	84.5 ± 4.8	4.5 ± 3.1	5.3 ± 3.7	5.5 ± 4.6
Maple	10.2 ± 6.5	57.1 ± 8.8	14.0 ± 4.5	18.8 ± 9.7
Mixed	51.4 ± 8.4	19.0 ± 5.5	9.0 ± 3.1	20.6 ± 8.5

^1^In descending order of basal area: *Quercus alba*, *Q*. *prinus*, *Q*. *velutina*, *Q*. *coccinea*, *Q*. *rubra*

^2^In descending order of basal area: *Acer saccharum*, *A*. *rubrum*

^3^In descending order of basal area: *Liriodendron tulipifera*, *Populus grandidentata*, *Carya* spp., *Nyssa sylvatica*, *Prunus serotina*, *Ulmus* spp.

Beds were encircled with wire attached to reinforcing bar at each corner at a height of approximately 13 cm. Bird exclusion netting was draped over the bed and wire and pinned to the ground around the plot to prevent additional litter input and loss. In the fall of each year, litter that had fallen onto the netting was removed. In the second year, two strips of 5x10 cm mesh fencing approximately 15 cm tall and 4 m long were added to the interior of each bed to prevent the netting from sagging, which had resulted in litter compaction. The partitions also helped reduce wind redistribution which had occurred within some plots. All beds in the study were sprayed with a 10 s misting of glyphosate herbicide in early summer of each year to minimize herbaceous growth and kill woody resprouts, which otherwise would have affected litter composition and microclimate. We do not know if glyphosate affects decomposition, but note that the amount applied was consistent across all beds.

Beds were sampled for analysis in April 2010 after 4 years of fall litter additions. We restricted analyses to data from spring 2010 in order to allow fuel beds to develop and because of early caging effects. Sampling consisted of 24 depth measurements of relatively undecomposed litter (L), partly decomposed litter that typically was present below the L layer (fermentation, F), and humus (H) across each bed and removal of two 25x25 cm sub-plots for which L-, F-, and H-layer depths were noted and layers individually bagged for dry mass determination. The H-layer was collected down to mineral soil. Layer definitions are from [48]. After dry mass determination, mineral mass mixed into the H-layer was determined by loss on ignition and subtracted to provide organic mass. Bulk density measurements from the sub-plots were multiplied by depth measurements to estimate loading by layer across the beds. F and H layers were combined (FH) for all analyses.

As a point of comparison for experimental fuels, we sampled bulk density of *in-situ* beds in spring 2010; that is, beds that developed adjacent to common gardens from the litter of surrounding trees. The basal area by species of surrounding overstory trees was estimated with a 10 factor prism [49] in two locations so as to encompass each common garden and adjacent forest. Trees were grouped into oak and hickory species and mesophytic species. Of interest was whether gradients in environmental variables and species composition were related to *in-situ* fuel bed characteristics in a way that was consistent with results from the common garden fuel beds. Linear, least-squares regressions were used to relate species composition to fuel bed characteristics.

### Spring dry-down fuel moisture

The dry-down period we sampled began with 18 mm of rainfall ending at 0400 on 22 May 2010. Starting on May 24^th^, 2010, the second day after the rain event, we began a daily sampling of L- and FH-layer moisture from each common garden bed. Sampling was conducted from noon through mid-afternoon through May 27th. Samples were collected in plastic bags which were then sealed and their wet masses determined soon after. Material was then dried at 50°C until no further mass loss occurred. Moisture contents are expressed as a fraction of dry mass. Moisture contents of L and FH layers were averaged for each type of plot (maple-dominated, oak-dominated, and mixed source) at each common garden location for the analysis. Meteorological conditions were monitored in a clearing at the VFEF headquarters (Fig 2).

### Thermochemistry and cone-calorimeter tests

In order to model fire spread and fireline intensity of the common garden fuel beds and more fully describe the combustion process, we estimated heats of combustion and other variables with cone-calorimeter tests. Further, we expected lignin fractions to be related to both decomposition and combustion and measured them through chemical analyses. Litter (L) layer samples were collected for thermochemical determinations from ridgetop and stream terrace topographic positions in beds with maple- and oak-dominated litter. We were limited in the number of samples we could process by time and cost and decided to focus on the landscape positions that we thought would be most different. In April 2010, two replicate 15x15 cm samples of L and FH material and underlying mineral soil were collected and wrapped in aluminum foil to maintain integrity. Though care was taken to avoid litter compaction, some compaction was unavoidable. Samples were transported to the US Forest Service’s Forest Products Laboratory (FPL) in Madison, Wisconsin, and cut down to 10 x 10 cm squares as required for the cone calorimeter. Heavy-duty aluminum foil was folded to cover each side of the sample up to 3 cm leaving the top open. Cone calorimeter samples and excess material for analytical tests were stored in the 80°F and 30% RH conditioning room at FPL until equilibrium moisture content had been achieved (verified by repeated weighing). Moisture content as a fraction of dry mass was determined for excess litter at equilibrium in the conditioning room prior to preparation for combustion by drying in a 50°C drying oven until no further mass loss occurred.

The L-layer material for analytical testing was ground with a Wiley Mill using a 20 mesh (2 mm) screen and mixed thoroughly. Ash, total carbohydrate, and total lignin fractions were determined in the analytical laboratory at FPL. Of particular interest for decomposition [20] and combustion was the lignocellulosic index (LCI):

LCI=LF(LF+CF) (1)

where *L*_*F*_ is the fraction of litter dry mass accounted for by total lignin and *C*_*F*_ is the fraction of litter dry mass that is total carbohydrate. Samples were dried in a vacuum oven at 45°C overnight for compositional analysis of cellulose sugars, Klason lignin (acid insoluble lignin), and acid soluble lignin (ASL). Standard FPL protocol was used to quantify lignin involving a primary hydrolysis of the dried 100 mg sample in concentrated sulfuric acid (H_2_SO_4_) followed by a secondary hydrolysis of the sample diluted to 4% H_2_SO_4_ in an autoclave at 120°C. The Klason lignin fraction retained after filtration of the hydrolysate was dried and it’s mass determined gravimetrically. Acid soluble lignin was determined with a spectrophotometer based on a method published in National Renewable Energy Laboratory (NREL) Analytical Procedure (NREL/TP-510-42618). Total lignin is Klason lignin and acid-soluble lignin combined. The sugar composition of the hydrolysate (arabinan, galactan, glucan, xylan, and mannan) was determined by High Performance Liquid Chromatography with pulsed amperometric detection (HPLC-PAD) with a Dionex ICS-3000 ion chromatograph system using the method described in [50]. Total carbohydrate was calculated as the sum of the yields of the individual sugars.

A cone calorimeter subjects a sample to a specified radiant flux (kW m^-2^) which ignites the sample and measures mass loss and gas evolution. We used a Model CONE2AutoCal, manufactured by Atlas Electric Devices Company of Chicago, IL, modified for additional measurement capabilities. A metal plate protects the sample from irradiation prior to test initiation. The methodology standards for the cone calorimeter followed in this study are ISO 5660-Part 1 (International Organization for Standardization 2002) and ASTM E1354 (ASTM International 2002). Gas analysers measured the oxygen, carbon monoxide, and carbon dioxide in the exhaust stack. A 41-mm orifice plate was used for a measured exhaust flow of 0.012 m^3^ s^−1^. Scan rate for measurements was 4 Hz. The samples were placed directly on the sample platform in its aluminum foil with no covering screen. Prior to testing, the depth of the combined L and FH layers was measured on each of the four sides of the sample to enable bulk density determinations.

The samples were irradiated at 35 kW m^-2^ and piloted ignition imposed. An irradiance of 50 kW m^-2^ of imposed heat flux is more typical for cone calorimeter testing. After ignition, approximately 12.5 kW m^-2^ was expected to be added to the flux arising from the heating elements in the cone resulting in a total flux to the sample approaching 50 kW m^-2^. Piloted ignition mimics ignition of the volatile stream in the presence of spreading flames. An irradiance of 25 kW m^-2^ was used in Dibble *et al*. [51] but was inadequate for this study because significant volatile mass from beds would have been lost before piloted ignition were achieved.

During the test, mass loss and oxygen consumption rates were determined and time to ignition was recorded. Heat of combustion and near peak heat release during the flaming period was calculated from oxygen consumption and mass loss. Because of our interest in actively spreading fires, we focus here only on results from the flaming combustion phase in cone calorimeter tests. We tested the effects of litter source and topographic position on the following variables: time to (piloted) ignition, peak heat release rate, and heat of combustion around the time of peak heat release rate.

### Fire spread index and modeled fireline intensity

Field and laboratory results were used to calculate a spread index of complex units that’s been shown to indicate a fuel bed’s propensity to carry a fire in beds whose properties overlapped those in this study [9]. The index was calculated for each topographic position and litter source combination during a five day dry-down period. The numerator of the index includes variables that relate to the total and rate of heat generation while the denominator describes the heat sink:

LCI=LF(LF+CF) (2)

where *h* is the flaming heat of combustion (kJ kg^-1^), σ is the fuel particle surface area to volume ratio (m^-1^), β is the packing ratio (dimensionless, based on ash-free bulk density and 400 kg m^-3^ fuel particle density), δ is fuel bed depth (m), and *Q*_*T*_ (kJ kg^-1^) is the total heat required to carry the fuel through pyrolysis (see details in [10]). Variables are specific to the L-layer. The exponents (a, b12, b3, and c) are constants from [10]. We used the spread index instead of spread probabilities derived from it because the index is more continuously distributed.

Modeled fireline intensity was calculated from litter loading, measured heats of combustion, and rate of spread from the Rothermel fire model [52]. Rate of spread was calculated using fuel bed, fuel particle, and combustion properties measured in this study. Conversion to SI units was aided by [53]. Moisture of extinction (sensitive to fuel properties as opposed to a constant moisture of extinction normally used) was calculated from a re-arranged Equation 8 in [9] and data from this study. Wind speed was set to a constant 8 km hr^-1^. Flaming heats of combustion estimated from cone calorimeter tests were used in rate of spread calculations along with surface-area-to volume ratios and ash contents averaged across plots of the same litter type. Fireline intensity is the product of heat of combustion, fuel consumption (assumed to be equal to L-layer loading), and rate of spread [54].

We did not have data for flaming heats of combustion and ash fractions for all treatment combinations and replicates for fire spread and fireline intensity analyses. As such, we used species average flaming heats of combustion from ridge and stream terrace landscape positions for maple and oak litter because we found only species effects on this variable. Mixed beds received the average of values from maple and oak beds. A constant ash fraction was used to calculate ash-free fuel loading for use in the spread index because values for species and landscape positions did not differ. Because fuel-moisture sampling was conducted at only three sites, only those three sites were available for analysis

### Statistical analyses

Each common garden was a randomized complete block with two replicates of each of three litter types averaged to provide a single replicate in each block for analysis. Common gardens were established at four topographic positions within each site (Fig 2). Statistical analyses were conducted with PROC MIXED in SAS [44]. Replication was balanced in all designs and Type 3 sums of squares were used as the basis for significance tests. Analyses were conducted for loading and bulk density of both L and FH layers, the lignocellulosic index, and combustion characteristics from cone-calorimeter tests. Separate mixed-model analyses of source effects were conducted for loading and bulk density that included both common garden (maple, mixed, and oak) and *in-situ* beds. Days since wetting rain was included as a repeated factor in analyzing dry-down moisture contents of L and FH layers. Repeated measures analyses were also conducted for spread index and fireline intensity for which moisture was a key variable. To account for the correlation between days we used the autoregressive AR(1) covariance structure where the current day’s value is related to the previous day’s value. Site was included as a random variable in all statistical models while topographic position and day were fixed effects. Levene’s test detected a violation of the homogeneity of residual error variance assumption among topographic positions in analyses of LCI and for all repeated-measures analyses (i.e., L and FH moisture, spread index, and fireline intensity over the dry-down). For these analyses, topographic position was treated as a grouping variable in the random statement. All dependent variables were natural-log transformed for analysis except for spread index and fireline intensity, which were rank transformed because of the large proportion of low values, particularly early during the dry-down period. The Kenward-Rogers method was used to determine denominator degrees of freedom. Differences among means were tested with Tukey’s HSD method which involves a multiple-comparisons adjustment for p-values and confidence limits [44]. Data used in analyses are provided as supporting information (S1–S8 Tables).

## Results

### Topographic variables

**Fig 3:**
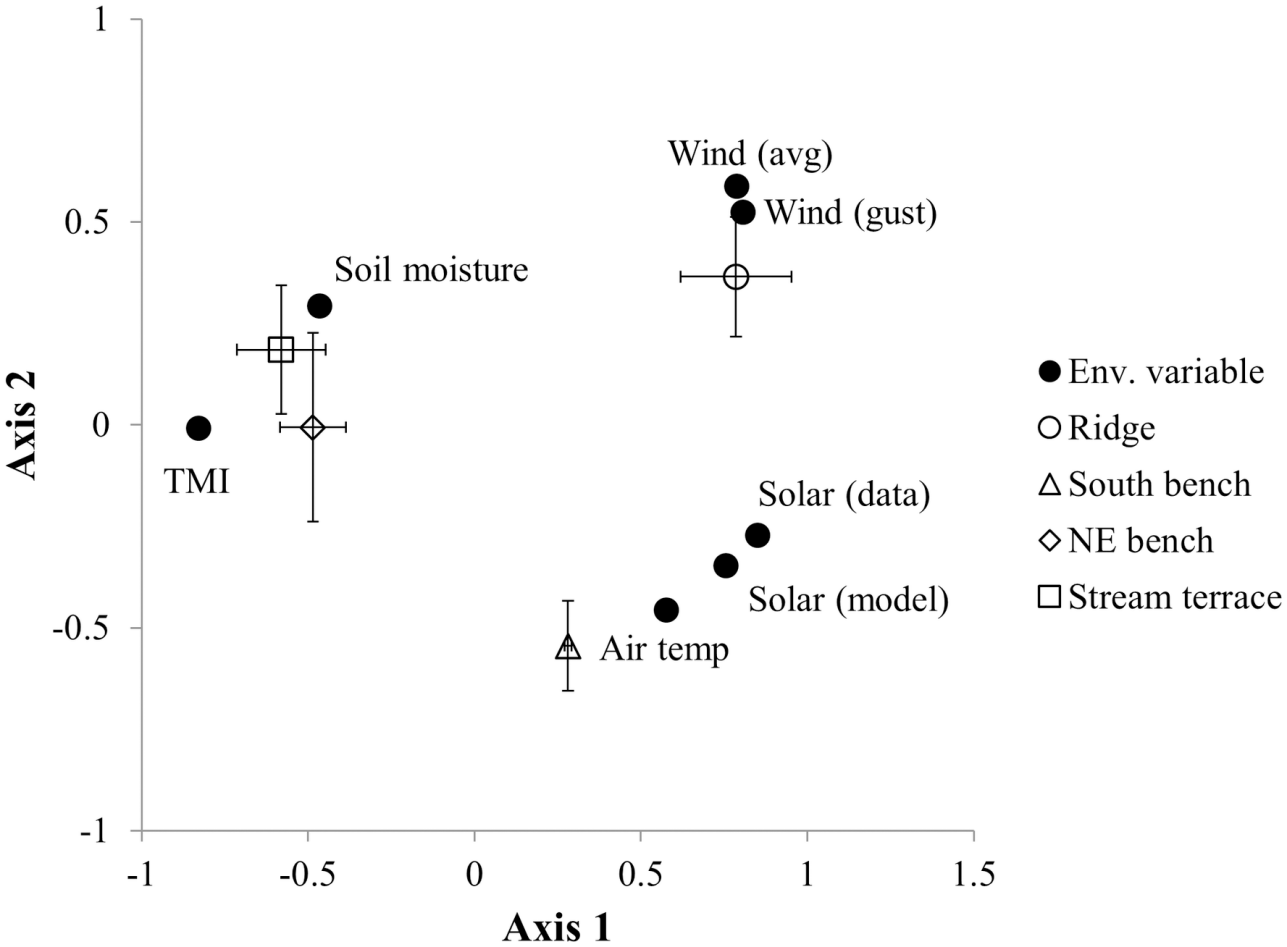
Factor analysis of modeled and measured environmental conditions at common garden locations. Included are factor scores for modeled and measured solar radiation, modeled soil moisture index, measured soil moisture, measured air temperature, and measured average wind and maximum gust speeds. Factor 1 explains 64% of the variation and 16% is explained by Factor 2. The average score and standard errors for each topographic position from N = 4 sites (Fig 2) are included in the plot. The standard error for south benches along axis 1 are small and not visible.

Topographic positions differed in environmental conditions and overstory composition. Relationships among factors for measured and modeled solar radiation and soil moisture and measured wind and air temperature are shown in Fig 3. Considering Factor 1 (which explains 64% of the variation), ridge positions, in particular, and south benches were associated with higher modeled solar radiation, wind, and air temperature, while northeast bench and stream terrace positions were associated with higher modeled and measured soil moisture. Overstory species composition showed a strong gradient of oak dominance on ridge and south bench positions and mesophytic species dominance on northeast bench and stream terrace sites (Table 2). As such, overstory species composition co-varies with topographic gradients. Differences in environmental conditions among topographic positions are indicated by error bars in Fig 3. Northeast bench and stream terrace sites are similar on average, but the errors indicate substantial variability, which may help explain the variation in species composition between these topographic positions (Table 2).

**Table 2:** Percentage of tree basal (mean ± standard error) area accounted for by different species groups for each topographic class where the common gardens were located.

Species Group	Ridge	South Bench	NE Bench	Stream Terrace
Oak^1^	71.4 ± 5.8	62.2 ± 16.2	26.6 ± 11.3	9.3 ± 6.7
Hickory	17.4 ± 3.3	3.9 ± 1.3	16.2 ± 4.8	6.8 ± 4.1
Maple^2^	3.4 ± 1.2	16.7 ± 7.0	14.7 ± 6.8	12.9 ± 6.1
Yellow-poplar	2.4 ± 1.5	11.9 ± 10.1	19.3 ± 7.4	34.6 ± 12.3
Other mesophytic^3^	0.0 ± 0.0	2.6 ± 2.6	17.2 ± 7.9	33.4 ± 8.7
Other^4^	5.4 ± 5.4	2.6 ± 2.6	6.0 ± 4.8	2.9 ± 1.0

Means for each topographic class are composed of data from N = 4 sites (Fig 2).

^1^ In descending order of abundance: *Q*. *alba*, *Q*. *velutina*, *Q*. *prinus*, *Q*. *coccinea*, *Q*. *rubrum*, *Q*. *muehlenbergii*.

^2^In descending order of abundance: *A*. *saccharum*, *A*. *rubrum*

^3^In descending order of abundance: *Fagus grandifolia*, *Tilia americana*, *Aesculus flava*, *Prunus serotina*, *Ulmus* spp., *Nyssa sylvatica*, *Carpinus caroliniana*, *Cornus florida*, *Juglans nigra*.

^4^*Pinus* spp., *Pinus strobus*, *Sassafras albidum*, *Oxydendrum arborea*

### Fuel bed mass and bulk density

**Fig 4:**
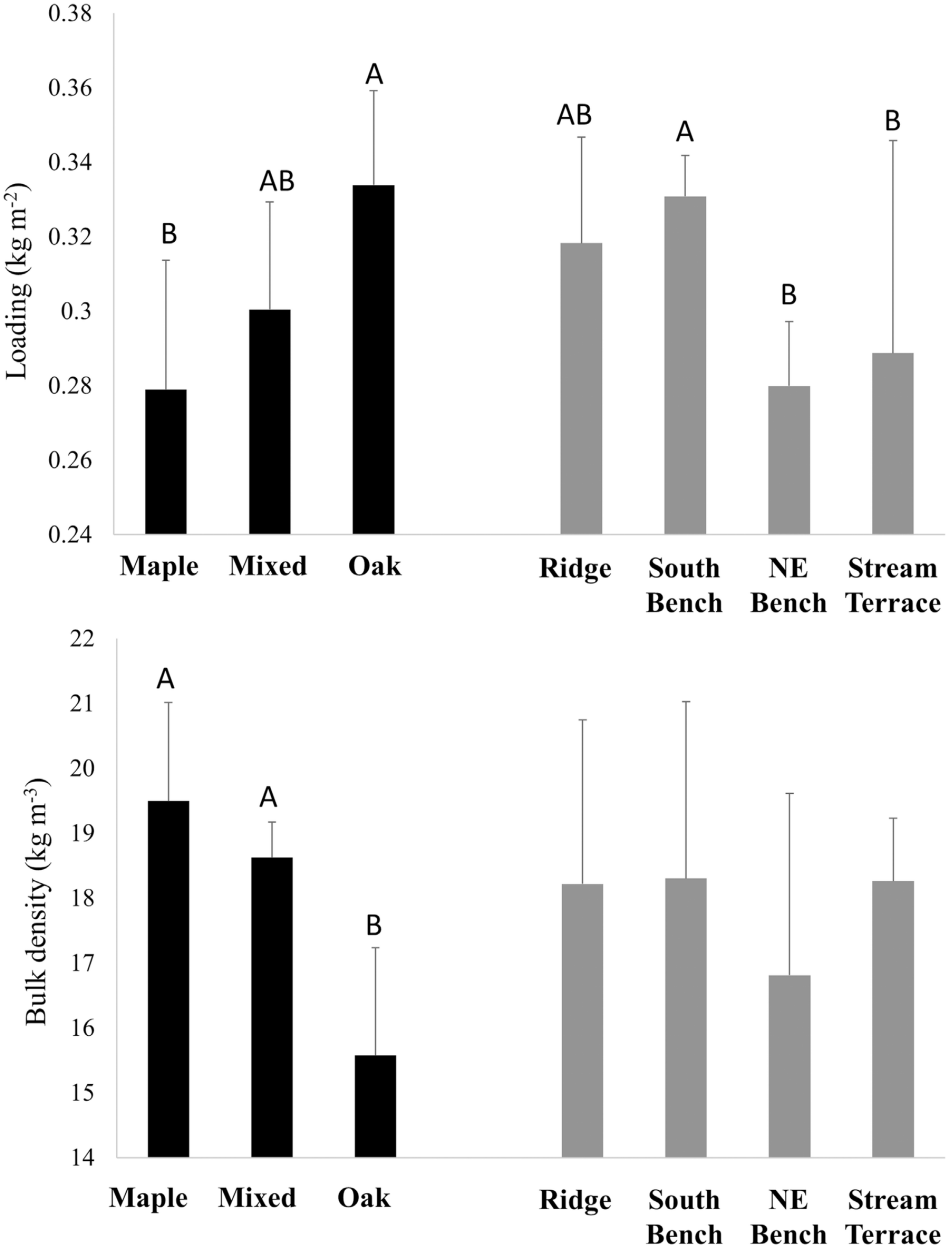
Common garden fuel loading and bulk density–L layer. Loading and bulk density by litter source and topographic position in common garden beds after four years of fall litter additions (see means in Table 3). Beds were sampled during the transition between winter and spring when most prescribed burning occurs. For significant main effects, differences among means were determined by Tukey’s HSD method and are indicated by different letters. Standard errors are shown.

**Fig 5:**
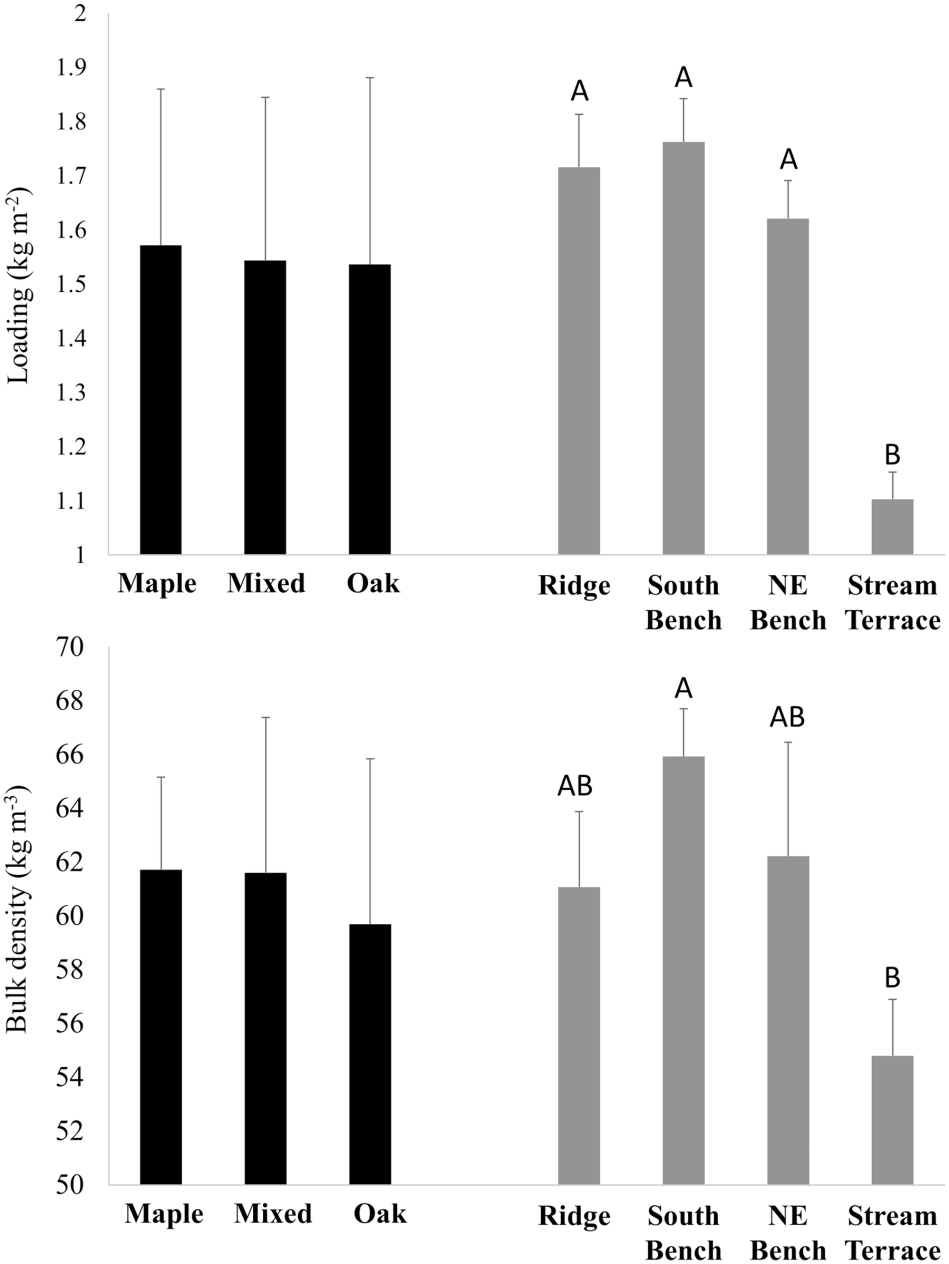
Common garden fuel loading and bulk density–H layer. Loading and bulk density by litter source and topographic position in common garden beds after four years of fall litter additions (see means in Table 3). Beds were sampled during the transition between winter and spring when most prescribed burning occurs. Only topographic effects were significant and differences among means were determined by Tukey’s HSD method and are indicated by different letters. Standard errors are shown.

In the mixed-model analysis of log-transformed common garden data, L-layer loading was significantly affected by both litter source and topographic position (P<0.001) but their interaction was not significant (P = 0.3). Litter (L) loading was greatest for oak, least for maple, and intermediate for mixed beds (Fig 4). Common gardens on northeast-bench and stream-terrace landscape positions had lower loadings than common gardens on south benches while ridgetop gardens were intermediate. Bulk density of the L layer was affected by litter source (P<0.0001), but not topographic position (P = 0.4), with oak beds having the lowest bulk density. In contrast, loading (P<0.0001) and bulk density (P<0.01) of the FH layer were both affected by topographic position but not by litter source (P≥0.7). The lowest FH loadings and bulk densities developed in common gardens on stream terrace positions (Fig 5).

**Fig 6:**
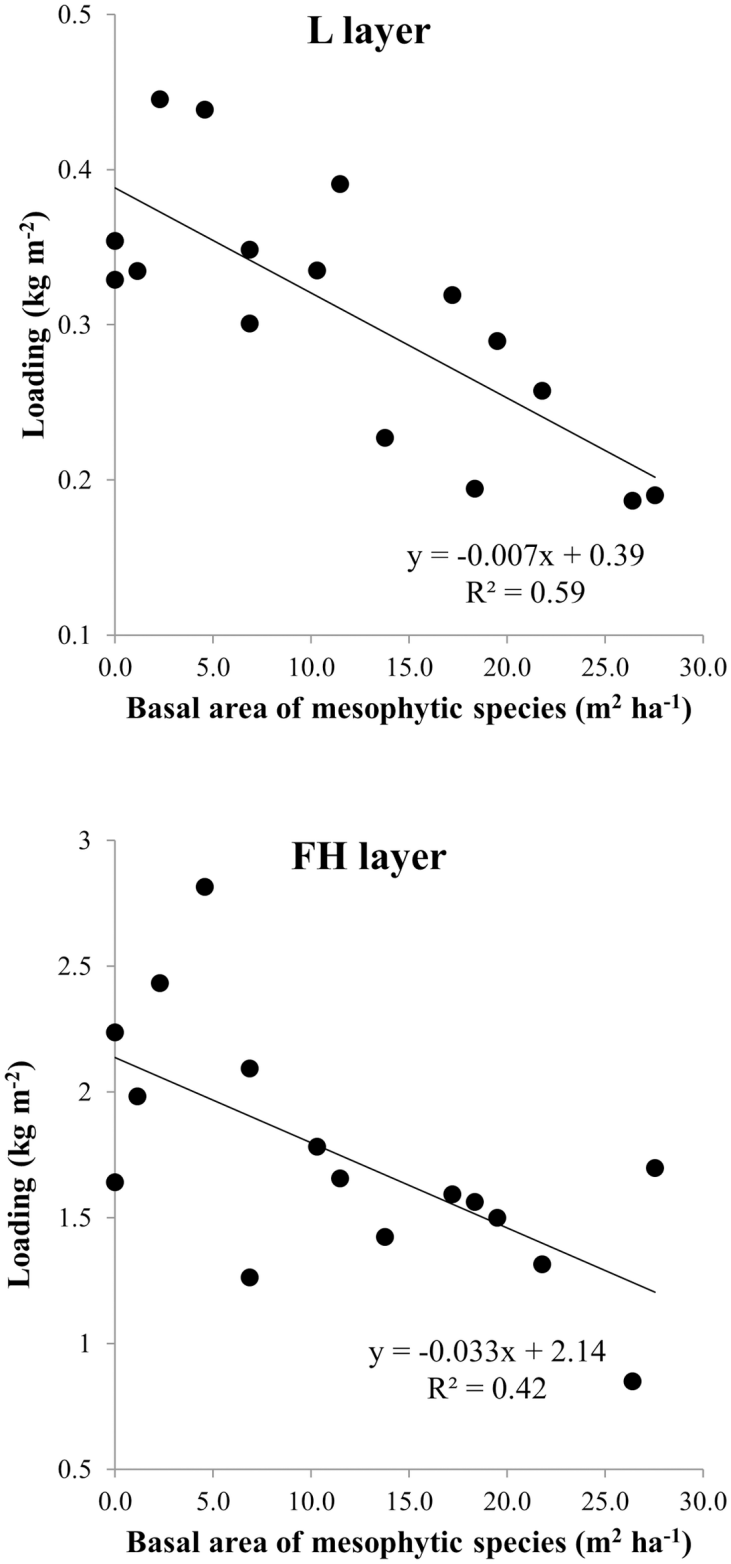
*In-situ* fuel bed characteristics in relation to overstory composition. Linear regression between the loading of L and FH layers for *in-situ* beds and the basal area of stems around common gardens that were mesophytic. *In-situ* beds were not manipulated in any way and were sampled adjacent to common gardens. Oak and hickory and mesophytic stems made up >90% of the species composition of common gardens (Table 2). As such, plots of loading as a function of oak and hickory relative abundances yield relationships that are nearly the inverse of those shown here.

In post-hoc contrasts among litter types, loading of the *in-situ* L layer was similar to that of common garden beds (Table 3). Loading of the FH layer did not differ among common garden beds and *in-situ* beds (as such, no contrasts are shown). Bulk density of the *in-situ* L layer was equal to that of oak-dominated beds but lower than that of maple-dominated and mixed beds. The bulk density of the FH layer did not differ among *in-situ* and common garden beds (as such, no contrasts are shown). Litter and FH-layer loading of *in-situ* beds was negatively related to the basal area of mesophytic trees (Fig 6). Bulk density of the *in-situ* L and FH layers was not related to overstory composition (P>0.05, results not shown), in contrast to the lower bulk densities of the oak L layer in common garden beds (Fig 4).

**Table 3:** Mean L- and FH-layer loading and bulk density on common garden beds and *in situ* beds after four years of fall replenishment.

Layer	Class	Loading (kg m^-2^)	SD	Difference (P<0.05)	Bulk density (kg m^-3^)	SD	Difference (P<0.05)
L	Maple	0.28	0.05	B	19.5	2.6	A
	Mixed	0.30	0.04	AB	18.6	2.6	A
	Oak	0.33	0.05	A	15.6	3.3	B
	*In-situ*	0.31	0.08	AB	16.3	4.0	B
FH	Maple	1.57	0.39		61.7	11.5	
	Mixed	1.54	0.37		61.6	9.9	
	Oak	1.54	0.44		59.7	11.8	
	*In-situ*	1.74	0.48		57.2	10.4	

Loading and bulk density were sampled during late winter/spring 2010 to reflect conditions in the main prescribed fire season in the region. *In situ* fuel beds developed from the litter of surrounding trees with no manipulation and were located adjacent to common gardens. Means and standard deviations (SD) by species source are shown. Replication is N = 16 in all cases and differences (indicated by different letters where a significant main effect of litter source was found) are determined by Tukey’s HSD method.

### Spring dry-down fuel moisture

**Fig 7:**
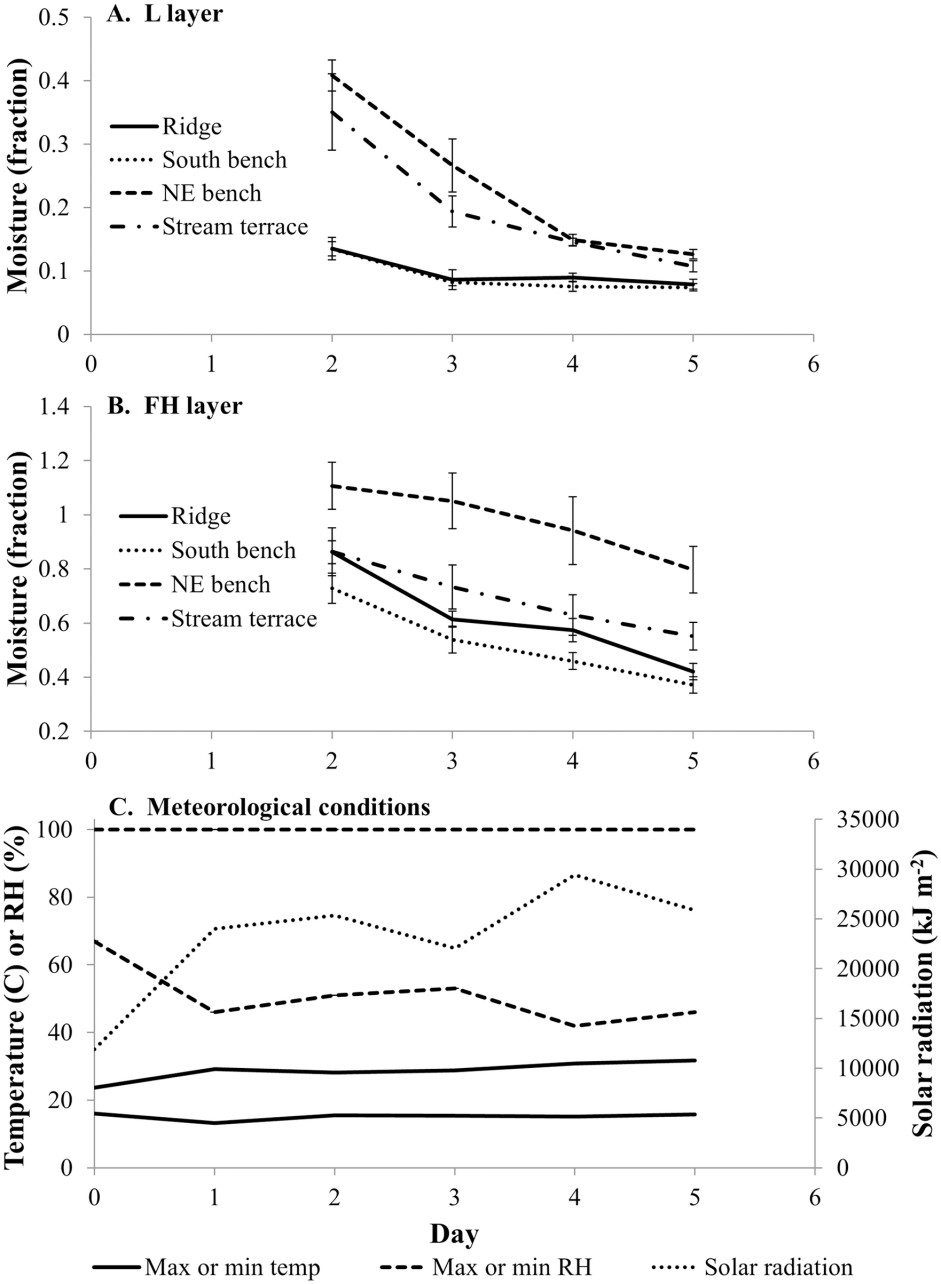
Fuel moisture of common garden beds and key weather variables over a five-day dry-down in May 2010. Effects of topographic position and day were significant and are shown while litter source effects were not significant and are not included (Table 4). Data are separated between the L and FH layers (panels A and B, respectively). Standard errors are included. Note that the scale for the FH-layer plot has a larger range because of greater moisture contents. Weather variables (C) were measured at the VFEF headquarters (see Fig 2 for location). A wetting rainfall occurred early on 22 May 2010 (day zero).

Litter layer moistures (expressed as a fraction of dry mass) were affected by topographic position, dry-down day, and their interaction but not by litter source (Table 4). Litter moistures were highest on northeast benches and stream terraces early in the dry-down (Fig 7A). Litter moistures became more similar among landscape positions as the dry-down proceeded (Fig 7A) explaining the significant interaction term. In contrast to the L layer, there was no significant main effect of topographic position on moisture of the FH layer (Table 4) despite northeast benches appearing to have the highest moisture contents. We can ascribe the lack of significance in part to low error degrees of freedom for the test after adjustments for inhomogeneous error variances associated with different topographic positions (e.g., see Fig 3). There was a significant interaction effect of day and topographic position on FH moisture but differences among topographic positions in drying trajectories appear to be subtle (Fig 7B). Trends in minimum and maximum temperature and relative humidity and solar energy from the VFEF headquarters weather station during the dry-down are shown in Fig 7C.

**Table 4:** The effects of species type, topographic setting, and day on measured fuel moisture over a 5-day dry-down in May 2010.

Layer	Effect	Df	F	P
L	Topography	3, 4.01	36.65	**0.0023**
	Species	2, 26.4	0.3	0.8
	Day	3, 70.2	54.3	**<0.0001**
	Topography x Day	9, 73.7	3.30	**0.002**
FH	Topography	3, 3.0	2.0	0.3
	Species	2, 26.4	0.06	0.9
	Day	3, 68.5	65.9	**<0.0001**
	Topography x Day	9, 72	2.17	**0.03**

Type 3 tests of fixed effects in this repeated-measures analysis are based on log-transformed data. Main and significant Type 3 interaction effects are shown. Separate analyses are shown for L and FH layers.

### Thermochemistry and combustion in the cone calorimeter

**Fig 8:**
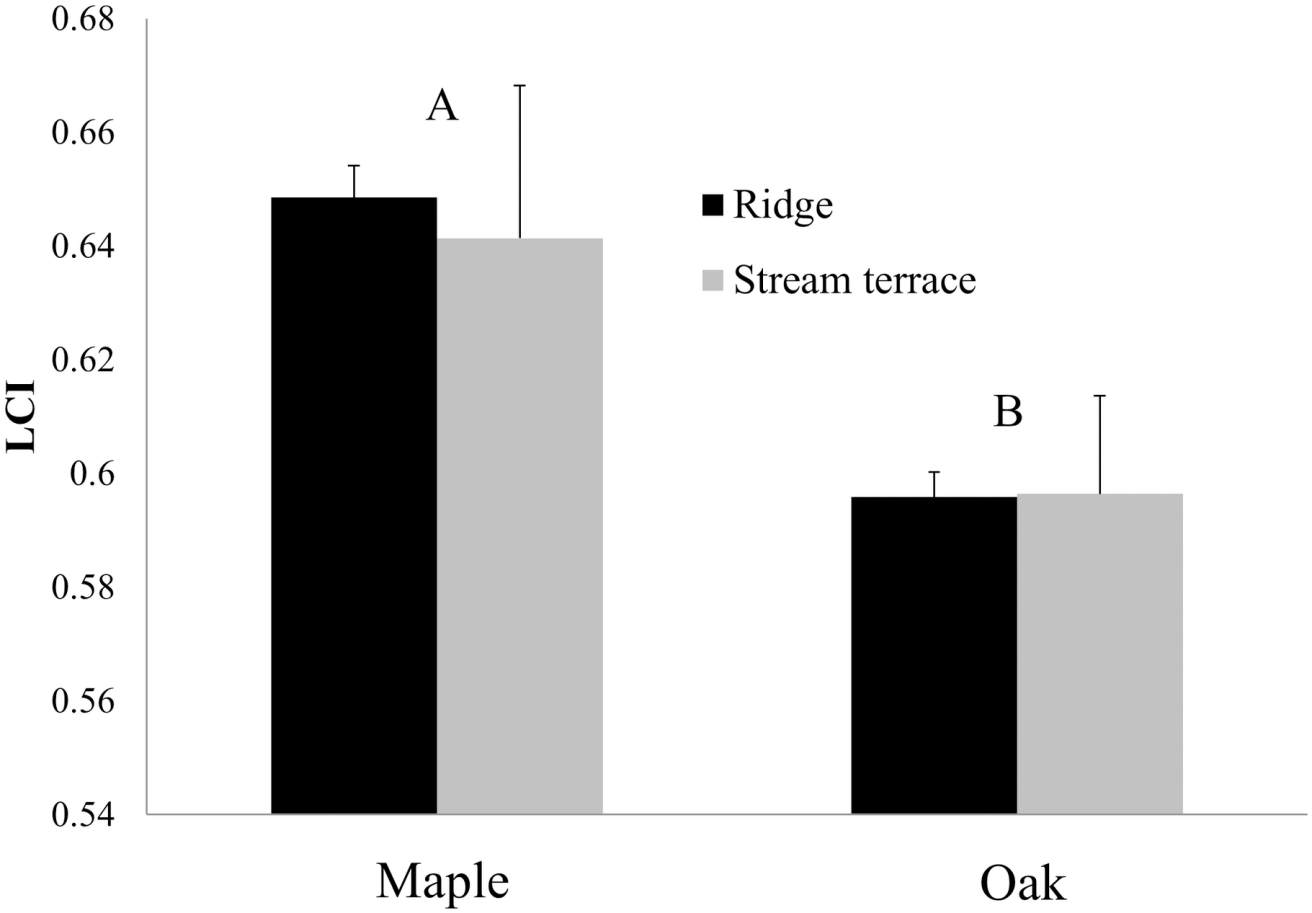
The effects of litter source and topographic position on the L-layer lignocellulosic index. Beds were sampled during the transition between winter and spring. Assays were conducted on L-layer samples only and included maple and oak beds but not beds composed of a mixture of maple and oak. A high lignocellulosic index indicates litter with high lignin content relative to total carbohydrates. Letters indicate the significant difference between species in post-hoc test by Tukey’s HSD method. Standard errors are shown.

Thermochemistry determinations were conducted on litter collected in spring 2010 from ridgetop and stream terrace landscape positions and for oak and maple beds only. Only litter source was significant in tests of main effects and their interaction. The lignocellulosic index (Eq 1) was highest for maple litter and least for oak (Fig 8), indicating a fuel with elevated lignin levels relative to cellulose. There was no significant difference among species or topographic position in acid-insoluble ash content of the litter layer (N = 16, ash fraction = 0.11).

**Fig 9:**
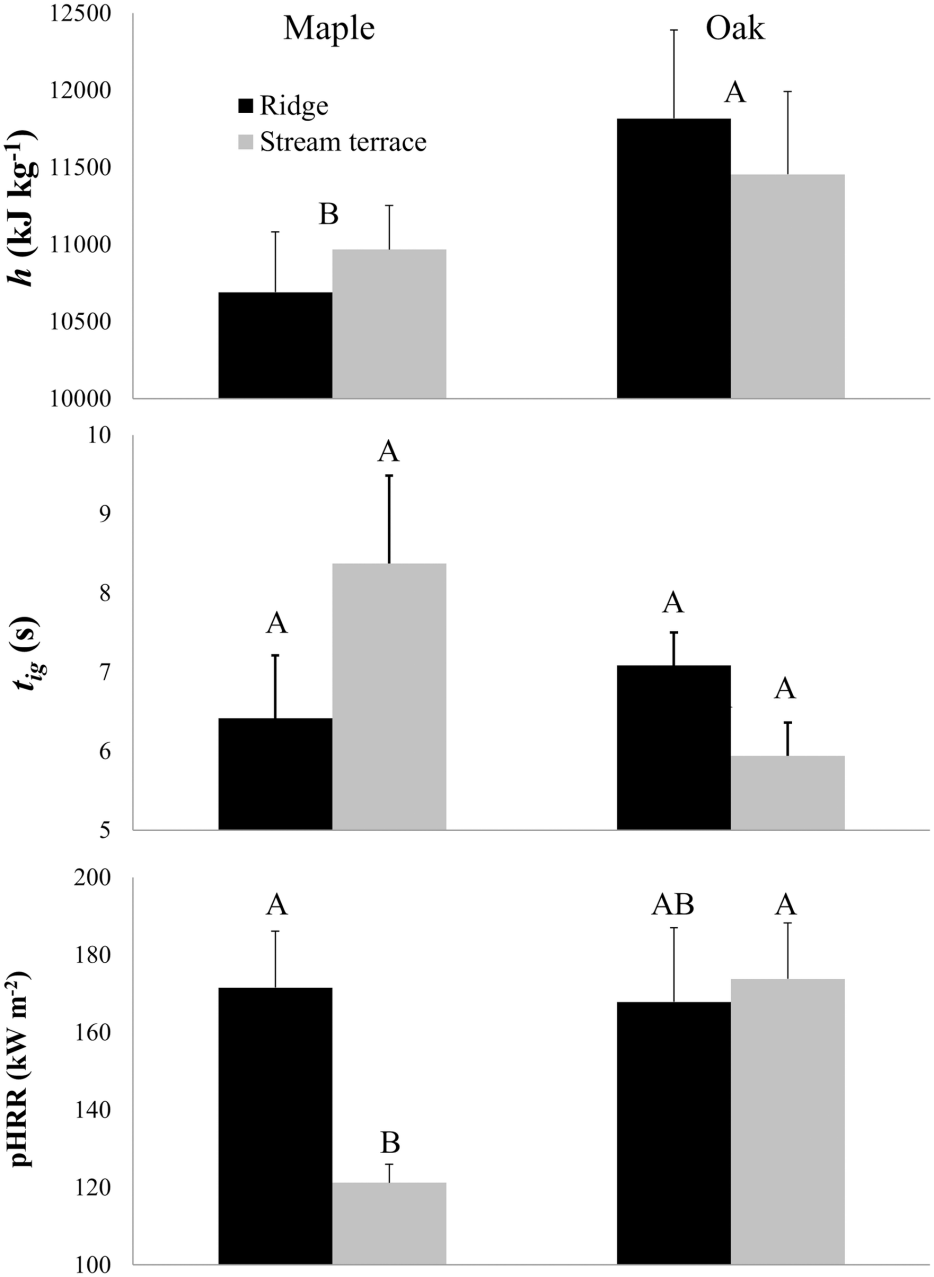
Effect of litter source and topographic position on combustion characteristics. Heat of combustion of the flaming phase (*h*), peak heat release rate (pHRR), and time to ignition (*t*_*ig*_) are shown for oak (left) and maple fuels (right) and for ridge and stream terrace landscape positions (see also Table 5). Combustion characteristics were determined by tests in a cone calorimeter on fuel bed samples and underlying mineral soil cut from plots in the spring after four years of fall litter addition. The variables primarily characterize flaming combustion of the (upper) L layer. Based on post-hoc test by Tukey’s HSD, letters indicate a significant difference between species for flaming heat of combustion or, where the interaction term was significant, among means of combinations of litter source and topographic position. Standard errors are shown.

In cone-calorimeter tests, flaming heats of combustion were only affected by litter source (Fig 9, top) while time to ignition and peak heat release rate were determined by significant interactive effects of litter source and topographic position (Table 5). Oak beds had higher flaming heats of combustion (12,600 kJ kg^-1^) than maple beds (10,400 kJ kg^-1^). The interactions affecting time to ignition (Fig 9, middle) and peak heat release rate (Fig 9, bottom) resulted primarily from long times-to-ignition and low peak heat release rates for maple in stream terrace landscape positions. Bulk density of the litter in samples used in cone tests did not differ by litter type as it did for litter in the field experiments.

**Table 5:** The effects of litter source and topographic position on fuel combustion characteristics (see also Fig 9).

Combustion characteristic	Effect	df	F	P
Flaming heat of combustion	Topography	1, 5.8	3.77	0.1
	Litter source	1, 7.1	6.47	**0.04**
	Interaction	1, 5.8	5.29	0.06
Ignition time	Topography	1, 6.47	0.37	0.6
	Litter source	1, 7.16	1.61	0.2
	Interaction	1, 6.47	9.67	**0.02**
Peak HRR	Topography	1, 5.7	7.84	**0.03**
	Litter source	1, 6.0	2.01	0.2
	Interaction	1, 5.7	19.19	**0.005**

Cone calorimeter tests were conducted on 10x10 cm samples excised from common garden beds of oak and maple origin from ridge and stream terrace topographic positions only. Type 3 tests of fixed effects are shown based on log-transformed data. Combustion effects include heat of combustion near peak HRR (kJ m^-2^), time to ignition (s), and peak heat release rate (HRR, kW m^-2^).

### Fire spread index and modeled fireline intensity

**Fig 10:**
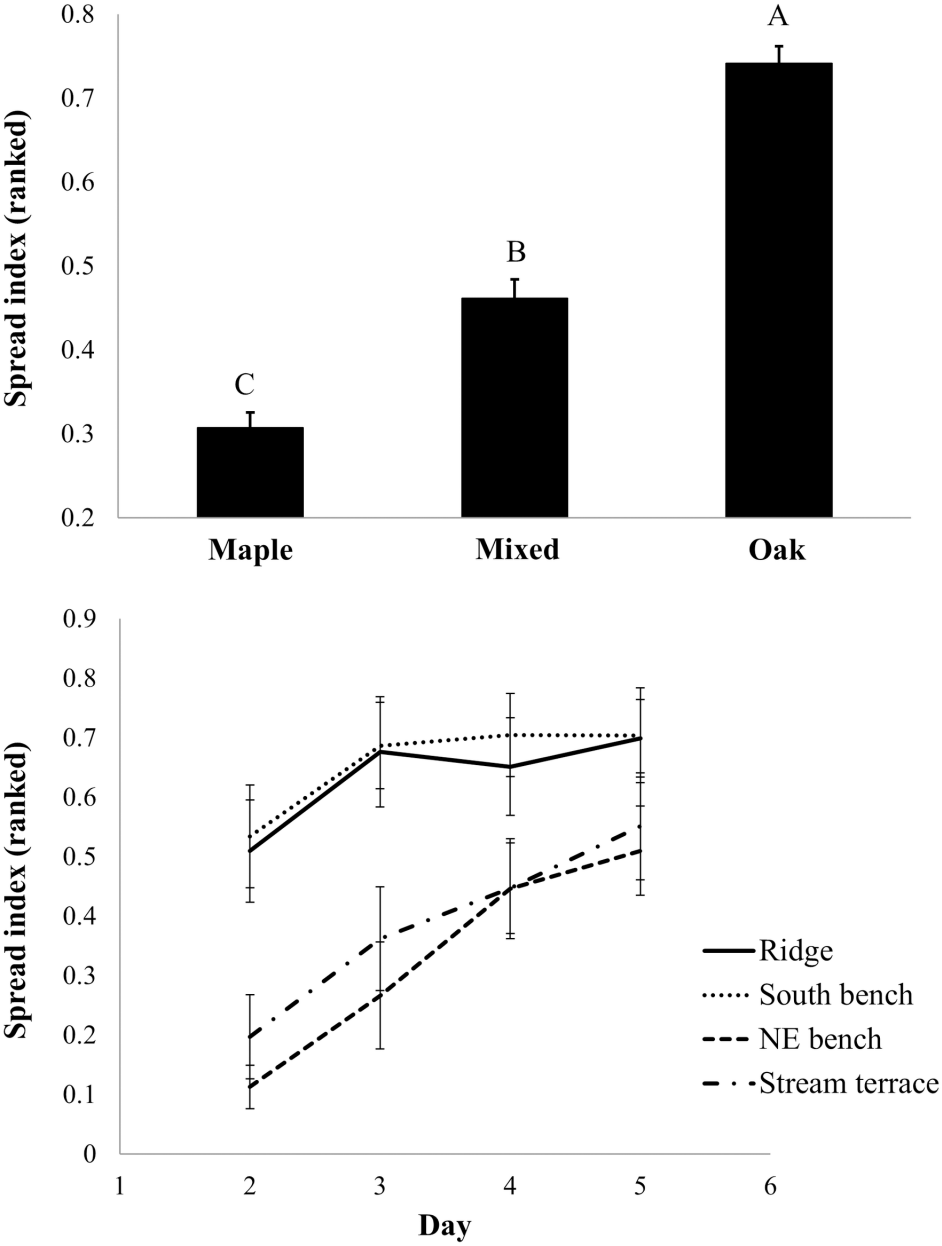
Fire spread potential for common garden fuel beds. Rank spread index is shown as a function of litter source, topographic position, and day during the dry-down. The spread index was parameterized with data from laboratory combustion trials [9] and applied to common garden fuel beds based on measurements of fuel bed, fuel particle, and combustion characteristics. Standard errors are shown.

**Fig 11:**
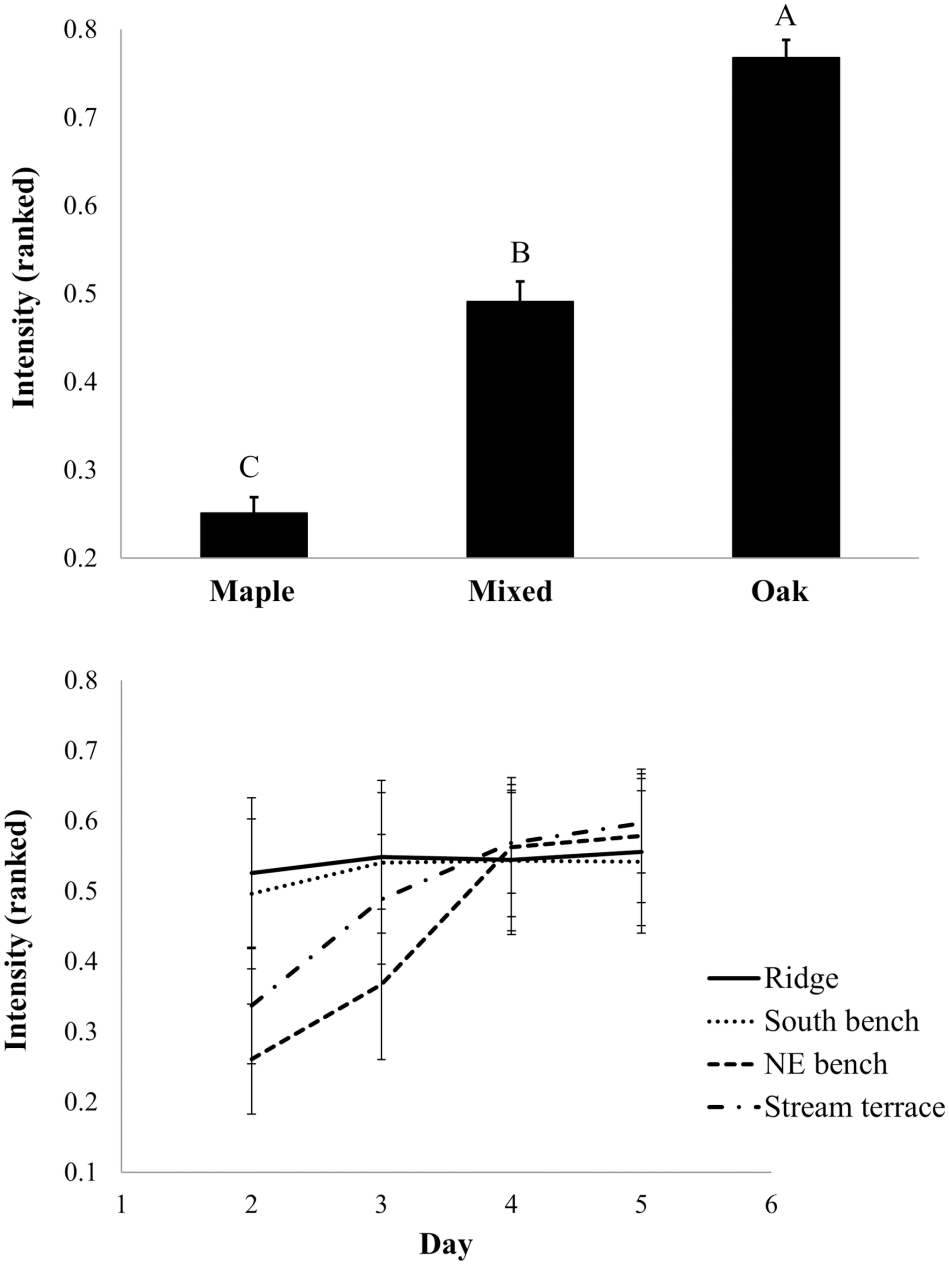
Modeled fireline intensity for common garden fuel beds. Ranked fireline intensity is shown as a function of litter source, topographic position, and day during the dry-down. Fireline intensity was calculated from [52] for common garden fuel beds based on measured fuel bed, fuel particle, and combustion characteristics. Standard errors are shown.

In the repeated-measures analysis of rank-transformed data, both fire spread potential and modeled fireline intensity were significantly affected by species and day (P<0.0001) across a 5-day dry-down period (Figs 10 and 11, respectively). Oak beds had the highest spread potentials and modeled intensities while mixed beds were intermediate. An interaction between topography and day (P = 0.02 and P<0.0001 for spread potential and fireline intensity, respectively) was the result of the more rapid dry-down of ridge and south bench landscape positions relative to northeast bench and stream terrace positions. Litter on ridge and south bench positions had dried considerably by day 2 (see Fig 7A). On wetter parts of the landscape, modeled fire spread and fireline intensity converged with that on ridge and south bench positions as the dry-down proceeded (Figs 10 and 11, respectively).

## Discussion

Results from the common garden experiment suggest that fuel beds (specifically, the L layer) derived from oak-dominated litter are more likely to carry a fire than beds derived from maple-dominated litter (Fig 10) and fires in oak-derived fuel beds are more likely to burn at greater fireline intensities (Fig 11). Topographic effects on the spread index and modeled fireline intensity were also significant with fire being less likely to spread and burn intensely on more mesic landscape positions early during a dry-down. The spread index [9–10] and modeled fireline intensity [52] are useful because they integrate a range of fuel bed (e.g., loading and bulk density, moisture), fuel particle (e.g., surface-area-to-volume ratio, ash content, lignocellulosic index), and combustion properties (e.g., flaming heat of combustion) in accord with physical properties and relationships governing fire spread. Our results show strong effects of species on fire spread and fireline intensity mediated by litter characteristics that are independent of topographic effects. Combustion results from cone calorimeter tests were more complicated: long times-to-ignition and low peak heat release rates were found for maple fuel beds, but only on beds that developed on stream terraces. These combustion results, however, are limited in that we tested beds from ridge and stream terrace landscape positions only. In answer to our central question, and considering the entirety of our data, both species and topography play important but largely independent roles in fuel bed development and potential fire behavior.

Patterns of loading that emerge from *in-situ* L and FH layers (Figs 4 and 5) are consistent with the effects of species and topography that we found in common garden beds (Fig 6). Overstory species composition and environmental gradients are correlated in our study area with higher basal areas of mesophytic species in sites with less solar radiation, higher soil moisture, and lower ambient temperatures (Fig 3). Sites with the greatest basal area of mesophytic species also had the lowest *in-situ* L and FH loadings. Our study is valuable in decoupling the separate effects of litter source and environment on fuel beds. In our common gardens, L-layer loading was reduced in beds formed from maple-dominated litter and also on north bench and stream terrace topographic positions. FH-layer loading was not affected by litter source, but was lowest on stream terrace positions. Accordingly, our data suggest that the patterns of loading of *in-situ* beds are determined jointly and independently by overstory composition and environmental conditions. We found no non-additive effects on decomposition of mixing litter from different species [24, 26], with our mixed-source beds showing intermediate values in L-layer loading and equal values in FH-layer loading relative to maple- and oak-dominated beds. Because of the co-variation between overstory composition and topography (Fig 6), our data could not be used to detect indirect effects of the overstory on litter beds resulting from overstory effects on decomposer communities and microclimates [22–23].

In contrast to Waldrop *et al’s* study in southern Appalachian forests [38] where the authors concluded that topographic variation in productivity of litter and woody material was largely balanced by concomitant variation in decomposition rates, we suspect that high litter productivity on mesic sites in the Ohio Hills was more than balanced by high decomposition rates in *in-situ* beds, resulting in lower L- and FH-layer loadings (Fig 6). Stottlemyer *et al’s* [37] finding of greater humus depths on xeric sites in the southern Appalachians is consistent with our FH-layer results. Our study and others [37–38] support the concept that fuels in dissected terrain in the central hardwoods must be understood in the context of topographically-determined gradients in solar radiation and hydrology that determine species composition and rates of productivity and decomposition and set the context for feedback processes. Our common garden study essentially controlled productivity rates by using a constant level of litter addition across the landscape in contrast to non-manipulative studies [37–38] whose results are affected by variation in productivity (but also have considered entire fuel complexes including downed woody and live biomass).

*In-situ* litter bulk density, in contrast to our common garden experiments where oak litter had the lowest bulk density, showed no variation along the joint gradient in topographic position and importance of mesophytic species (results not shown). A lack of differences in bulk density of *in-situ* litter suggest that bulk density differences may have been an experimental artifact, perhaps associated with the greater propensity for mesophytic species’ litter to fragment during collection, storage, mixing and redistribution. Despite care in avoiding litter fragmentation, a proportion of litter was in fragments (5%, 21%, and 19%, of litter by mass for oak, mixed, and maple beds in fall 2008, respectively, see Table 1) and would likely have reduced bulk density proportionally [13]. The order in fragmentation (oak < mixed = maple) is roughly the same order of L-layer bulk density found in common-garden beds (Fig 4). We explored the consequences of reduced bulk densities of maple-dominated litter for modeled spread potentials and fireline intensity by replacing bulk densities measured in beds of different kinds of litter with the average bulk density for all plots in each common garden. The effects of the substitution were subtle and, from this, we conclude that the species effect on bulk density was less important for modeled spread and fireline intensity than other characteristics such as loading (Figs 4 and 5), fuel moisture (Fig 7), lignin content (Fig 8), and thermochemistry (Fig 9).

*In-situ* litter may also have differed from common garden litter beds in the way litter beds are replenished during the fall. Maple leaves abscise earlier in the fall than oak leaves which, in mixed stands, would result in some degree of layering of the litter beds. More readily decomposable maple litter may often be present underneath oak litter and, as a result, become more compressed and remain more moist and prone to decomposition. Though we did not layer maple and oak litter, an indirect effect of fragmentation might have been increased decomposition rates of maple and mixed fuels through increased surface area on which decomposer communities could act. Despite differences in fragmentation and layering, we find that the results of our experiments were consistent with *in-situ* beds in that beds formed predominantly from maple and other mesophytic species’ litter have lower L-layer loadings by the time the spring fire season arrives (compare Figs 4 and 6).

A lack of a species effect on dry-down moisture dynamics (Table 4, Fig 7) contrasts with [29] who found reduced drying rates among mesophytic species relative to pyrophytic oaks and other fire-dependent species. Kreye *et al*. [29] proposed that beds dominated by mesophytic litter not only impede fire and reduce its fireline intensity, but also facilitate regeneration of mesophytic species in part because underlying soils would remain more moist. As such, [29] proposed that differences in drying behavior were a mechanism for mesophication. Our results show that drying dynamics of our fuel beds in the Ohio Hills were controlled by topography (Table 4, Fig 7). Moisture dynamics in the field are governed not only by drying rates (as shown in [29]) but also by the moisture contents towards which fuel beds trend (i.e., equilibrium moisture contents) under a given set of environmental conditions [55–56]. Our results suggest that the drying dynamics of L and FH layers in the field respond strongly to topographic gradients in environmental conditions (e.g., wind, insolation, soil moisture, air temperature and relative humidity; Fig 3) and secondarily, if at all, to litter source and related differences in such factors as fuel bed structure. Though the L layer of fuel beds composed of maple in our study had higher bulk densities (and thus might be expected to dry more slowly), there were no species differences in FH-layer loading or bulk density (Fig 5), which may help explain the overall lack of a species effect on dry-down dynamics.

Extrapolating from laboratory to field conditions may be problematic relative to dry-down dynamics in the same way that it can be problematic to extrapolate laboratory-based flammability studies to the field [57]. At the same time, conclusions from our dry-down sampling should be treated with caution because we sampled one dry-down event under conditions that were not entirely representative of late-winter/early-spring prescribed fire conditions. Though fires in May are likely to be more effective in controlling oak competitors, fires are not typically prescribed past April in the region currently because of concerns about increasing forest use by bats and other wildlife as the weather warms [58]. Dry-down events in April tend to occur before trees leaf out and are accompanied, often, by high forest floor insolation and higher wind speeds, particularly on ridge and south-facing slopes (Fig 3). Our May dry-down sample occurred when the leaf out period was well underway. The minimum relative humidities during the dry-down (≥40%, Fig 7C) were also on the high side of the range for mid-day relative humidities during prescribed-fires in the region [47, 59]. Perhaps lower humidities and more rapid dry-down of the L layer would expose species differences in Ohio Hills fuel beds, but the strong effects of topography we encountered suggest that it is unlikely that species effects would ever overshadow the effects of large topographic gradients in the conditions that determine moisture dynamics (Fig 3).

We measured loading, bulk density, and moisture for F and H layers separately. The F and H layers were combined for analyses because their properties were similar relative to expected effects on fire dynamics. We would expect the FH layer to support smoldering rather than flaming combustion [60]. The FH layer was more strongly affected by topography than litter source. In particular, topographic position was the only significant effect on FH-layer loading, bulk density, and moisture. Specifically, fuelbeds on stream terrace sites developed low loadings and bulk densities probably owing, in part, to the higher moisture contents (along with northeast benches) on those sites (Fig 7). We speculate that the majority of the reduction in FH layer loading on stream-terrace landscape positions was the result of consumption by non-native earthworms [61].

We were not able to burn plots in the field and, instead, relied on models and laboratory tests to assess the likely effects of fuel variability on combustion. A limitation of our cone calorimeter tests is that fuel bed samples derived from oak- and maple-dominated litter did not differ in bulk density even though, in the common garden beds, oak-derived beds had lower bulk density (Fig 4) and, thus, would be expected to burn even more intensely than they did in our cone calorimeter tests [62]. The packaging and transportation of samples apparently compressed the fuel beds to a similar bulk density. As well, moisture contents of maple and oak litter were not different. As such, cone calorimeter results (Fig 9, Table 5) showing higher flaming heats of combustion around the time of peak heat release rate for oak beds and long times-to-ignition and low peak heat release rates for maple fuel beds that developed on stream terrace slope positions must be related to inherent differences among fuel particle properties. A potential reason for litter source effects is the higher lignin content relative to carbohydrate found in maple-derived fuel beds in the spring (Fig 7). Fresh maple litter in the fall is known to be of high quality as a substrate for the decomposer community and high decomposition rates result in litter during spring that is relatively enriched in lignin (and depleted in cellulose, a high quality energy source for decomposers). High lignin contents are known to lead to greater stability during thermal decomposition [27] and, thus, an increased propensity for smoldering.

We used modeled fire spread (which incorporates rate of spread predicted from the Rothermel fire model [52]), litter loading from common garden beds, and heat of combustion from laboratory measurements), to assess potential differences in fireline intensity among fuel beds and landscape positions. Although we believe that the relative rankings of the potential for intense spread are reasonable, absolute values are suspect. Modeled fireline intensities from our common garden beds were often near zero because of high moistures, high bulk densities, and low loadings but also because of a known tendency for the Rothermel model to underpredict rate of spread and fireline intensity [63]. Our choice of values for heat of combustion would have further reduced spread rate and fireline intensity because we used measured effective flaming heat of combustion (12,600 and 10,400 kJ kg^-1^ for oak and maple fuels, respectively) rather than the higher low heat of combustion that was used in the development of the Rothermel model (18,600 kJ kg^-1^). In contrast, the moistures of extinction (moistures at which 50% of fuel beds would be predicted to not carry a fire) we calculated from [10] and the spread index (Eq 2) averaged 27% (with 5% standard deviation), a value that is similar to Rothermel’s [52] value of 30% applied to a range of fuel types. There has been much effort in recent years to develop more physically-based fire spread models [64–65] but none yet provide a fire behavior prediction system that is as sensitive as the Rothermel model to a wide range of fuel bed, particle, and combustion properties. Improvements in modeling tools will support future studies like ours.

We did not consider topographic variation in wind in modeling fire spread and fireline intensity. Ridges, in particular, and also south-facing slopes experienced higher average wind speeds during the study (Fig 3) because prevailing winds are from the southwest [66] and benches on northeast-facing slopes and stream terraces were sheltered topographically. Wind not only increases the intensity of fires burning with the wind [52] but also increases fuel drying rates [55–56]. As such, higher winds on ridge and south-facing slopes during the fire season would have direct and positive effects on fire spread probabilities and intensities. Increased drying rates would also translate into reduced decomposition rates on ridge and south-facing landscape positions, a result consistent with higher L-layer loadings (and lower decomposition rates) we found in our study on those landscape positions (Fig 4).

We compared fuel load and bulk density of beds in our common garden with *in-situ* litter beds in the same locations as common-garden plots in order to assess whether there was evidence for experimental artifacts from caging and litter fragmentation. During the first year, the bird netting that we suspended over the top of plots sagged and caused compaction of common garden beds. We prevented this effect in subsequent years by adding spacing material to the interior of plots. At the end of the experiment (the spring following four years of litter addition), bulk densities were similar between oak plots and *in-situ* litter but bulk densities of maple and mixed beds were higher than *in-situ* litter (Table 3). We assume that caging (even with no contact between netting and fuel beds) had a tendency to increase bulk densities across bed types. There was no obvious drifting of litter across plots after the first year when spacing material was added and we speculate that any subsequent compaction effect was owing to a suppression of litter redistribution, perhaps from a local dampening of wind gusts.

Because of known and substantial redistribution of litter down slopes in mixed-oak forests [39], we were restricted in establishing common gardens on relatively flat sites: ridges, mid-slope benches, and stream terraces. Accordingly, we were unable to fully explore topographically-determined gradients in soil moisture and incident solar radiation. For example, sites that are probably dryer than ridgetops (steep south-facing slopes) are common but were unavailable for experimentation. Extrapolating from our result that ridges and south benches exhibited the highest loadings and, thus the lowest decomposition rates, we speculate that south slopes, because they are hydrologically drier, would have exhibited further reductions in decomposition rates. Lower decomposition rates and litter subsidies from upper to lower slope positions might create an additive effect on fireline intensity as fires burn more intensely in beds with higher loading and upslope due to greater pre-heating of fuels [52].

We preferred sampling in spring over other seasons because prescribed burning is most commonly done in spring in this region while wildfires show a bimodal distribution, with wildfires most common in the spring and fall [67]. Fires late in the year before leaf-fall would burn in fuel beds with lower loadings and more advanced decomposition than early season fuel beds [12, 68]. Fires in late-season droughts are known anecdotally to consume substantial amounts of humus by smoldering combustion and cause high levels of tree mortality. In contrast, spring fires in the region often burn over moist FH layers and soil and tend to primarily support flaming combustion [69]. Our results from *in-situ* beds showing higher loadings in the FH layer on dryer landscape positions suggest that fire effects may be expected to be more acute on dryer parts of the landscape during fall, drought-associated wildfires.

The ecology of fuels [1] in oak-hickory forests on the unglaciated Allegheny Plateau in southeastern Ohio is a story of fire feedbacks set in the context of topographically-determined gradients in solar radiation, site moisture, soil fertility, nutrient cycling rates, and vegetation composition [31, 33]. Because of the independent effects of litter source and topography on fuels (Fig 4), the dominance of mesophytic species on mesic landscape positions in our study has resulted in substantially reduced L-layer loading on mesic landscape positions (Fig 6). The combined effects of species and topography suggests a mechanism for the inhibiting effect of topographic roughness on fire frequency found by [70] in Missouri. Counteracting the covariation of tree species composition and topography, frequent historical fires promoted oak dominance even in mesic landscape positions [7, 71]. Whether the dominance of fire resistant and resilient oaks has arisen because of historical fire or topography, our study suggests that the strong effects of litter source on fuels and fire behavior would reinforce oak dominance in a frequent prescribed fire regime [32, 72]. Our results suggest that the more difficult endeavor is to shift vegetation composition towards oak in topographic settings where mesophytic fuels impede fire spread and reduce fire intensities and, thus, limit injury and mortality of fire-sensitive trees.

## Conclusions

The results of our common garden fuels study and associated *in-situ* litter sampling suggest that fuel beds derived from oak-dominated litter are inherently more likely to carry fires and burn at higher fireline intensities than beds composed of litter dominated by maple foliage. At the same time, and largely independent of species composition, beds on more mesic landscape positions are less likely to carry fire and, if they do, would tend to support lower intensity fires than beds on xeric landscape positions, particularly during the early days of dry-down events. An implication of our results is that, with continued increases in the abundances of mesophytic species [7], prescribed fire may become less effective at maintaining oak dominance where oaks are present in the overstory but are failing to regenerate because of the shading by abundant mesophytic species. Overstory composition develops in interaction with the topographic gradient in the Ohio Hills ecosystem with fire creating positive feedbacks between robust fuel beds generated from oak litter on drier landscape positions and the dominance of oak species with fire-tolerant traits. Future studies focused on understanding and simulating central hardwoods ecosystem dynamics and disturbance interactions would benefit from further work on the effects of litter chemistry on combustion behavior, assembly and dynamics (in moisture and biomass) of litter beds in the field, and physics-based modeling of fires in litter beds.

## Supporting Information

S1 Table Common garden locations. Decimal latitude and longitude are provided for common gardens (S1.csv). Datum used was WGS1984. Site identifier and topographic positions (TOPO; 1 = ridges, 2 = south benches, 3 = northeast benches, and 4 = stream terraces) are indicated.
(CSV)

S2 Table Leaf litter collection sites. The decimal latitude and longitude of each of three replicate leaf litter collection sites where maple-dominated (LITT_CODE = 1) and oak-dominated (LITT_CODE = 3) litter were collected each fall are provided (S2.csv). Datum used was WGS1984.
(CSV)

S3 Table Modeled solar radiation and topographic moisture index for common gardens. Plot averaged values are provided in file S3.csv for modeled solar radiation (SolarRad, annual sum, MJ m^-2^) and the topographic moisture index (TMI). Sites and topographic positions (TOPO; 1 = ridges, 2 = south benches, 3 = northeast benches, and 4 = stream terraces) are indicated for common garden locations.
(CSV)

S4 Table Litter depth (cm) and loading (kg m^-2^) on common garden fuel beds. Litter depth and loading are averaged for analysis over two replicate beds in each common garden (S4.csv). Sites, topographic positions (TOPO; 1 = ridges, 2 = south benches, 3 = northeast benches, and 4 = stream terraces), and litter type (LITT_CODE, 1 = maple dominated, 2 = mixed, and 3 = oak-dominated) are indicated. Data are separated by forest floor layer (1 = L layer; 2 = F layer, and 3 = H layer). F and H layers were combined for analyses.
(CSV)

S5 Table Fuel moisture during the dry-down event. Fuel moisture is provided as a fraction of dry mass (M_FRAC). Date, sites, topographic positions (TOPO; 1 = ridges, 2 = south benches, 3 = northeast benches, and 4 = stream terraces), and litter type (LITT_CODE, 1 = maple dominated, 2 = mixed, and 3 = oak-dominated) are indicated for each common garden bed (S5.csv). Data are separated by forest floor layer (1 = L layer; 2 = F layer, and 3 = H layer). F and H layers were combined for analyses. Data are provided by individual bed with the designation (from 1 to 6) relating moisture contents to actual beds identifiable in our raw data. Data from the two replicate beds for each litter type in each common garden were combined for analyses.
(CSV)

S6 Table Thermochemical characteristics of maple- and oak-dominated fuels. Fraction of a 100 g sample that was Klason lignin (K_lignin), acid-soluble lignin (ASL_1_), acid-insoluble ash (AI_Ash_2_), and various sugars (arabinan, galactan, glucan, xylan, and mannan) is provided (S6.csv). Total carbohydrate is the sum of sugar values while total yield is the sum of all values. Replicate measures are provided for individual common garden beds at all four sites and for ridge (TOPO = 1) and stream terrace (TOPO = 4) positions and for maple- (LITT_CODE = 1) and oak-dominated beds (LITT_CODE = 3). Data were averaged for analysis across the two beds of each litter type in each common garden.
(CSV)

S7 Table Cone calorimeter combustion characteristics. Heat of combustion during the flaming period (FHC, MJ kg^-1^), time to ignition (tig, s), and peak heat release rate (pHRR, kW m^-2^), are provided for individual maple- (LITT_CODE = 1) and oak-dominated beds (LITT_CODE = 3)(S7.csv). Beds were located at all four sites and on ridge (TOPO = 1) and stream terrace (TOPO = 4) positions. Data were averaged for analysis across the beds of each litter type in each common garden. Combined L, F, and H bulk density (LFH_DENS, kg m^-3^) and the density of the underlying mineral soil (SUB_DENS, kg m^-3^) are also provided.
(CSV)

S8 Table Spread index and modeled rate of spread and fireline intensity. Ranked spread index (RankSI), modeled rate of spread (RankROS), and fireline intensity (RankI) are provided as used in analyses (S8.csv). Site, topographic position (TOPO; 1 = ridges, 2 = south benches, 3 = northeast benches, and 4 = stream terraces), and litter type (LITT_CODE, 1 = maple dominated, 2 = mixed, and 3 = oak-dominated) are indicated. Day since wetting rain is also provided.
(CSV)
